# Effect of Rule Modifications on Kinematic Parameters Using Maturity Stage as a Moderating Variable in U-10 Football Players

**DOI:** 10.3390/s24082462

**Published:** 2024-04-11

**Authors:** Francisco Javier García-Angulo, José Manuel Palao, José María Giménez-Egido, Enrique Ortega-Toro

**Affiliations:** 1 Department of Physical Activity and Sport, Faculty of Sport Science, University of Murcia, 30720 Murcia, Spain; franciscojavier.garcia19@um.es (F.J.G.-A.); josemaria.gimenez@um.es (J.M.G.-E.); 2 Sport Performance Analysis Association, 30720 Murcia, Spain; palaojm@gmail.com; 3 Department of Health, Exercise and Sport Management, University of Wisconsin-Parkside, Kenosha, WI 53144, USA

**Keywords:** competition, sports, cinematic variables, youth players, moderation analysis, football

## Abstract

The aim of the present study was to analyse the effects of regulatory modifications in competitive situations on cinematic variables, considering maturity stage as a moderating factor, in youth football players. A quasi-experimental study was conducted in which 45 players with a mean age of 9.47 ± 0.54 participated. The independent variable analysed was the modification of rules (playing time, scoring, and specific rules). The dependent variables analysed were cinematic variables. These variables were recorded with Wimu^TM^. The maturity stage was considered a moderating factor in this effect. The main results indicate that the modified competition reduced the total distance covered, maximum acceleration speed, and distance covered in acceleration and deceleration in different speed zones. In addition, the maturity stage was found to moderate the effect of the intervention on the total distance covered, distance covered by accelerating in zone 3, and distance covered by decelerating in zone 3. Thus, the proposed modification appeared to reduce the physical demand for competition. Furthermore, it reduced the differences between players with early maturational development and those with late maturational development.

## 1. Introduction

The new sport teaching methodologies defend the nonlinearity of sports systems; conditioning the subjects themselves; and the environment, the task, or other elements, such as feedback or methodology [1,2,3]. In this sense, modifications have been made in the training sessions and in competition itself. The effects produced by the two learning environments may be different.

In teaching sessions, the main modifications made refer to modifications related to the playing space [4,5], number of players [6,7], ball [8,9], or structural elements [10]. In general, these studies indicate that the use of constraints adjusted to the needs of athletes stimulates sport learning, increasing variability, number of actions, and intensity of movements.

Thus, in football training situations, the game space has been modified, showing that playing in large spaces (30 × 25; 24 × 36; 40 × 29) produces greater speed, distance, and number of sprints; a higher heart rate; and a higher spatial exploration index [11,12,13,14], besides encouraging amplitude and headers in wide spaces (43 × 47 m) [15]. Another common constraint in training situations is the modification of the number of players. In this way, playing in an inferiority situation (4 vs. 4; 4 vs. 6) promotes a greater number of sprints [11,16], and playing with more players (6 vs. 6) promotes a more durable possession [17]. Finally, regarding goal constriction, positioning goals in the corners increases the total distance travelled, the amplitude, and the distance to the centroid [18,19], while modifying the number and size of the goals from one standard goal to three small goals. It also increases the stretch index and efficiency [20,21].

On the other hand, the importance of rule modifications in competition has been shown in other sports. In this sense, the main modifications made are to the playing space [22,23]; the ball [23,24]; structural elements such as net height [10,25] and basket height [26,27]; and several elements such as net height, court size, and serving rules [28]. The most significant results indicate that competition is a formative environment that needs to be modified to offer better learning opportunities [22]. Thus, modifying the goals by adjusting them to the players’ abilities increases performance and self-efficacy, while modifying elements such as the height of the net or the type of ball allows for a more dynamic game and affects the effectiveness of the serve [23,25].

The effects of different constraints on football competition were also analysed. These studies can be divided into those that analyse the effect of constraints on technical–tactical aspects and those that analyse conditional aspects.

Studies that analyse tactical–technical aspects have been found to modify several constraints. In this way, a comparison in the U-12 stage was made between playing in an 8 vs. 8 situation in a 58 × 38 m space with 6 × 2 m goals and a 5 vs. 5 situation in 38 × 20 m with 3 × 2 m goals. It was found that the 5 vs. 5 situation increased the variability of actions and interactions of the goalkeeper and outfield players [29,30]. Other studies have analysed the effects of changes in the number of players and goals on decision making in technical–tactical actions in U-9 players, showing that situations with fewer 3 vs. 3 and 5 vs. 5 players and smaller goals (1.65 × 5 m) increase decision-making, improve technical efficiency, and result in fewer missed passes [31,32].

Studies have been conducted on modifying rules in competition and analysing conditional aspects. In this sense, it has been analysed how the modification of the offside rule, modifying the offside line from the midfield to the goalkeeper’s area line, affects the heart rate, distance covered, and speed of the players [33]. In contrast, the modification linked to increased substitutions (from three substitutions to five) in professional players causes players to increase the distance covered and the distance covered at high speed. This allow players to maintain physical performance [34,35].

Neither studies analysing the effect of constraints in teaching sessions nor those analysing constrictions on competition have taken the effect of maturational age into account. In this sense, there is a large amount of scientific evidence that states that the level of maturity stage influences technical aspects such as ball control, dribbling, or passing [36]; physical aspects such as endurance, strength, and flexibility [37]; and executive functions [38]. Thus, only the study by Birrento et al. [39] was found. This work shows that basketball players at an early maturity stage have better cinematic performance than players at a late development stage.

The aim of the present research is to analyse the effect of rule modifications in competitive situations on cinematic variables, considering maturational age as a moderating factor, in youth football players.

## 2. Materials and Methods

The study initially involved 52 subjects belonging to four federated teams in the FFRM. This sample was reduced to 45 due to the fact that the goalkeepers were not analysed and to the experimental abandonment (*n* = 2). The final sample of 45 players had a mean age of 9.47 ± 0.542. The players trained at least twice a week and competed at a regional level [40]. The legal tutors of the participants were informed of the purpose of the study and signed an informed consent form, allowing participation in the study. This study was approved by the ethics committee of the University of Murcia (M10/2024/024).

A quasi-experimental A-B study was designed. Situation “A” was an 8-a-side football tournament under FFRM rules, and situation “B” was a tournament where the rules were modified. The same teams and players participated in both tournaments. The number of players (8 vs. 8), the playing area (58 × 38 m), the goal size (6 × 2 m), and the ball size (4) were stable between the two tournaments (Table 1).

The independent variable in this study was the tournament rules. In this modification of the rules, the organization of the playing time was modified, changing the two traditional 25-min periods to five independent 10-min periods. In addition, the scoring was modified, eliminating continuous scoring and adding isolated scores in each set (2 points for winner, 1 point for draw). Player substitutions were modified, requiring all players to participate in a minimum of two sets and prohibiting them from playing three consecutive sets. Substitutions were prohibited during the periods, except in the last period. The goalkeeper played at least one set as an outfield player. In addition, owing to concerns about heading at early stages [41], direct headers were banned, requiring the ball to bounce in order to head it. Another rule imposed was to increase the distance from the defenders at the goal kicks to the offside line (thus increasing the distance between the ball keepers and defenders).

For the selection of dependent variables, we used those presented in the scientific literature on youth football players [42,43,44,45,46,47]. In this way, the variables of distance covered, distance in speed sections, number of accelerations and decelerations, maximum acceleration and deceleration speed, maximum speed, and player load were selected (Table 2). Only the effective playing time was analysed.

Wimu^TM^ inertial devices (Realtrack Systems, Almeria, Spain) were used for data collection. This device is composed of four accelerometers, a gyroscope, a magnetometer, and a GPS, among other sensors. The inertial devices had a frequency of 1000 Hz. This device is valid for measuring the variables under study. More specifically, it reports validity for the variables linked to distance covered (intraclass correlation coefficient [ICC]: 0.97), speed (ICC: 0.94), and player load (ICC: 0.99) [48,49,50]. The data recorded by these devices were processed using SPRO software version 1.00.989.

In relation to the dependent variables that presented cutoff points or reference values, the scientific literature used values similar to those of adult players. Therefore, it was necessary to establish cutoff points and reference values adjusted for players in the Benjamin stage. Consequently, two-stage clustering using Euclidean distance was performed to define the cut-off points for the variable’s velocity, acceleration, and deceleration (Table 3).

The data were recorded in two tournaments (three days between the two tournaments). Both tournaments were played simultaneously and under similar weather conditions. In addition, an extra day prior to the tournament was used to inform parents of the aim of the study and collect informed consent information. At this meeting, the height and weight of the players and the height of the father and mother were recorded to estimate the maturity stage of each player. Measurements were recorded using a commercial portable stadiometer (Tanita BF-522W, Tokyo, Japan).

Each tournament consisted of six matches; thus, information from the twelve matches was recorded. The order of the matches was the same for each tournament. To reduce the influence of fatigue, a minimum rest period of 15 min was imposed between matches in which the same team participated. For the tournament with federated regulations, a substitution system was established with the intention of distributing the minutes in a balanced way. This measure was not necessary in the modified tournament, because the set and substitution rules ensured a balanced distribution. The teams were given 15 min to warm up. Their coach conducted this warm-up. It was requested that it be the same warm-up as the one used in the federated competition. No limitations were imposed on the coaches’ feedback.

There was a time gap of approximately 15 min between each match. This period was used for the players to warm up and apply the protocol for setting up recording devices. Under the indications of this protocol, the measuring devices were fully charged before the start of each tournament, and the devices were started 15 min before the start of each match.

Once the devices were recording the activities, they were placed on the players. At the end of each match, the devices were removed from the players and recharged before the next match. Each inertial device and player had a code so that the players’ data could be associated with the data on the devices.

After each tournament, the data were extracted from the devices by associating the data file code with the player code, thus constructing a data matrix per game consisting of all players in those matches. Once this matrix was constructed, the effective playtime data were selected.

### Statistics

The normality of the data was calculated using the Shapiro–Wilk test, reporting all variables as normal, except for the variables of maximum deceleration speed, acceleration distance in zone 2, and acceleration distance in zone 3 (Table 4).

To calculate the differences between each tournament, the Wilcoxon paired samples test and Student’s t-test were used, and the effect size was calculated using the biserial rank correlation for the non-normal variables (r < 0.1): 1 no effect/very small effect, r ≥ 0.1 ≤ 0.3 small effect, r ≥ 0.3 ≤ 0.5 medium effect, r ≥ 5 large effect) and according to Cohen’s d for normal variables (d ≤ 0.2 small effect, d > 0.2 ≤ 0.6 medium effect, d > 6 ≤ 1.2 high, >1.2 very strong) [51].

Bayesian analysis was also performed using Poisson’s scheme. To interpret this analysis, the Bayes factor BF^p^_10_ was calculated, which was interpreted according to the following scale: <1/100 support of extreme evidence H0, 1/100 to <1/30 support of very strong evidence H0, 1/30 to <1/10 support of strong evidence H0, 1/10 to <1/3 support of moderate evidence H0, 1/10 to <1/3 support of moderate evidence H0, 1/3 to <1 support of ambiguous evidence H0, 1 to 3 support of anecdotal evidence H1, >3 to 10 support of moderate evidence, >10 to 30 support of strong evidence H1, >30 to 100 support of very strong evidence H1, and >100 support of extreme evidence H1 [52].

A within-participant repeated-measures moderation analysis was used to test whether the maturational age of the players affected the effects of the rule change (differences between the two tournaments). The relationships between the predictor (federated tournament variable) and outcome (modified tournament variable) were examined by testing the interactions between these variables and the stable moderator variable (W1) using a simple moderation model [53]. Thus, an ordinary least squares regression analysis was conducted using SPSS macro MEMORE v2.1 [53]. Due to the continuous nature of all variables, interactions were analysed using the Johnson–Neyman procedure to identify areas of significance where the intervention was significant according to maturational age [53].

## 3. Results

Table 5 lists the data related to normal cinematic variables. It can be seen that there are statistically significant differences in total distance, maximum acceleration speed (t = 10.76, *p* < 0.001), distance covered decelerating in zone 1 (t = 9.449, *p* < 0.001), distance covered decelerating in zone 2 (t = 9.4, *p* < 0.001) and distance covered decelerating in zone 3 (t = 818, *p* < 0.001). Similarly, anecdotal evidence was found for total distance; very strong evidence for distance covered decelerating in zone 3; and extreme evidence for maximum acceleration speed, distance covered decelerating in zone 1, and distance covered decelerating in zone 2 regarding the effects of BF^p^_10_.

**Figure 1:**
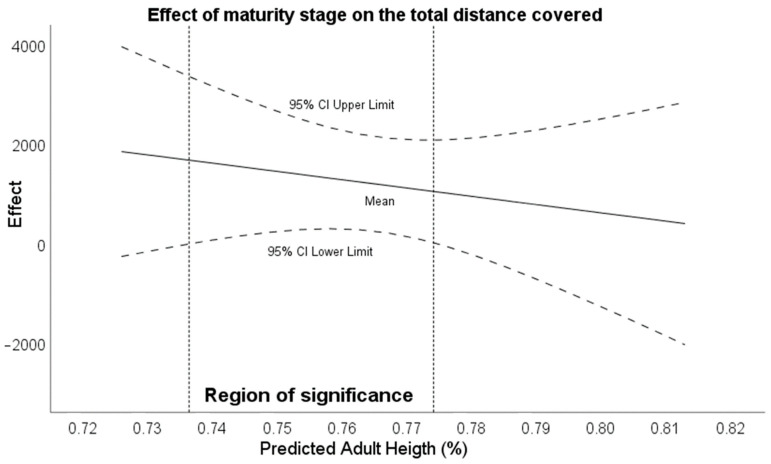
Effect of maturity stage on total distance covered.

**Figure 2:**
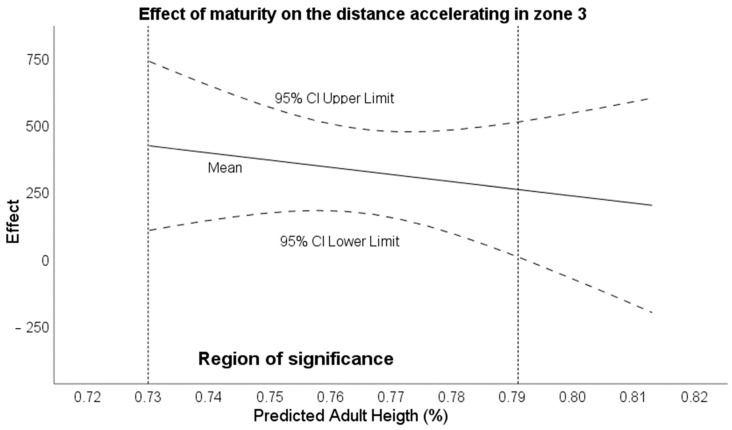
Effect of maturity stage on acceleration distance covered while accelerating in zone 3.

Regarding the moderator analysis, the Johnson–Neyman procedure identified that there were ranges of maturity stages that conditioned the total distance covered between tournaments. Thus, it was observed that with a maturational stage between 73.6% and 77.4%, there were significant differences for the distance variable (Figure 1). Moreover, in the deceleration distance variable in zone 3, the range of significance was between 72.81% and 79.14% of the maturity stage (Figure 2). For the rest of the variables in Table 5, no transition points of significance were found where age was a moderator of the variables.

Table 6 shows the variables with non-normal distributions of the data. The descriptive and inferential data are presented in this table. Thus, differences between tournaments were observed for the maximum deceleration speed (W = 990; *p* < 0.001), reflecting the BF^p^_10_ extreme evidence of the tests. Regarding the acceleration variables, significant differences were only found in the acceleration distance in zone 3 (W = 889, *p* < 0.001), and extreme evidence support was reported.

**Figure 3:**
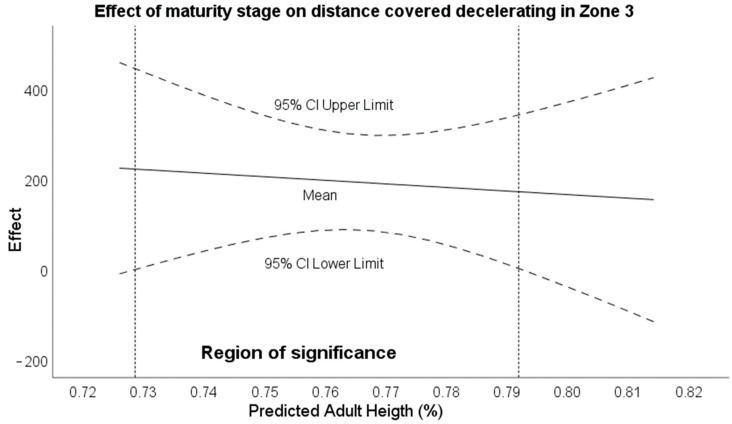
Effect of maturity stage on deceleration distance in zone 3.

Regarding the transition points where maturity was a moderator of the differences, for the acceleration distance variable in zone 3, it was estimated that there were maturity stage effects in the range below 79.16% of maturation (Figure 3). No specific range was found for the maximum deceleration speed (the entire maturity range analysed had an effect).

## 4. Discussion

The aim of the present study was to analyse the effects of regulatory modifications in a competitive situation on cinematic variables, considering the maturity stage as a moderating factor in youth football players. In this sense, significant differences were found; specifically, the modified tournament reduced the total distance covered, maximum acceleration speed, deceleration distance in zone 3, and acceleration distance in zone 3, while it increased the deceleration distance covered in zones 1 and 2 and the maximum deceleration speed.

Regarding the effect of the maturity stage, it was found that the maturity stage moderated the effect of the intervention on the total distance covered, accelerating distance in zone 3, and decelerating distance in zone 3. In addition, it was found that in the late maturational stage, the maturity stage moderated the effect of the intervention on the variables of maximum sprint speed, maximum speed accelerating, maximum speed decelerating, and distance decelerating in zones 1 and 2.

These results seem to indicate that the modification of the rules reduces the physical demands. In this sense, the results reflect that the proposed modification moves physical values away from the absolute stage model, giving importance to other formative aspects of sport, as indicated in the academic literature [54]. In this way, it allows the focus of the teaching–learning process to be placed on other behaviours apart from the physical aspect, such as decision making and perceptual aspects [55,56,57].

Similarly, it is necessary to consider the risk of injury to young players during sports practice. In particular, in young players, a high risk of injury has been found during competitive situations [58]. Furthermore, there is a correlation between high physical demands in sport and the number and degree of sports injuries [59]. Therefore, taking our results into account, the proposed modification reduces the physical demands of young athletes by addressing the aspects that increase the risk of injury.

On the other hand, addressing the effect of regulatory modifications on the maturity stage, the modification of the regulations proposed in this study reduces the differences between players with early and late development, showing a particular effect on players with late maturational development.

In this regard, special concern has been shown for adjusting competitions to offer the same learning opportunity. Under the current competition system,, it has been shown that, in the formative stages, players with early maturational development are captured at a higher percentage by talent development programs than players with late development, giving special importance to physical development in this selection process [60]. For this reason, several academic proposals have emerged that advocate for competition based on biological age.

In this way, academic proposals have emerged that advocate competition on the basis of biological age. Thus, Arede et al. [61] justified the importance of conducting competitions based on maturational age as it reduces the physical demands. The results found in this study are consistent with the findings of our study.

In this sense, the proposed competition seems to have a greater impact on players with late development, decreasing their physical demands. This allows players to explore different behaviours, increase variability, and have the same chances of success, thus favouring their efficiency, self-efficacy, enjoyment, and adherence to sports practice [1,62]. These variables mentioned above are related to sporting success and health aspects [63].

Regarding the injury rate, young players with late development report higher injury rates than players with early development [64]; for this reason, it is necessary to give special attention to this kind of player in the adjustment of competitions.. Thus, the proposed rule modification reduces the number of accelerations and decelerations in zone 3, which is related to a lower injury rate, as distance covered, accelerations, and changes in pace are considered some of the main injury mechanisms in young players [65,66]. In this way, young players can take part in a less damaging sport.

This study has great practical implications for the design and adjustment of competitions at the formative stage. Thus, it has been justified that the proposed modification reduces physical demands and decreases the differences between players with early development and those with late development. These data should be considered by sports federations when designing competitions. Competitions should thus be adopted that reduce the differences between players with early and late maturational development. In this respect, it is recommended that federations and competition organizers advocate for the implementation of the competition proposed in this study. Regarding coaches and sports clubs, it is recommended to structure youth teams by maturity age and not by chronological stage. In this way, the teaching–learning processes can be adjusted to their abilities.

The main limitation of the study was that, due to the time available for data collection, the players did not perform any training practice with the modifications posed, so the experience may have affected their physical performance in both tournaments. Finally, conditioned by the structure of the sports teams, it was not possible to carry out a randomized controlled study design due to the fact that each team included players without being able to randomize those teams.

In view of these limitations, future research should analyse the effects of these regulatory modifications from a holistic perspective, taking into account tactical–technical, physical, and psychological aspects. Hence, studies should be carried out that analyse physical, technical–tactical, and psychological variables of the same modification. In addition, because the instrumentation used is expensive and difficult to operate, studies can be performed with other devices that are less expensive [67]. The effect of maturational age should also be analysed under other regulatory modifications to search for a type of competition that minimizes the effects of age. In this sense, studies should be carried out in competition to unify the biological age of the participants.

## 5. Conclusions

Sports in the formative stage should be adjusted to the teaching–learning process of players, taking into account their abilities and skills. Thus, sports in early stages should move away from physical performance and focus on technical–tactical learning while reducing the risk of injury. In this way, the proposed tournament reduces the physical demands and differences in cinematic parameters between players with early and late maturational development, thus creating a useful learning environment for all players regardless of their maturational development.

## Figures and Tables

**Table 1:** Regulatory aspects established in both competitions.

	FFRM Federated Tournament	Modified Tournament
Number of players	8 vs. 8	8 vs. 8
Playing space	58 × 38 m	58 × 38 m
Size of goal	6 × 2 m	6 × 2 m
Size of the ball	4	4
Duration	2 × 25 min	5 × 10 min
Score	Total goals scored	Sets won
Substitutions	Free	Without changes (except 5th set)
Special rules	---	-The goalkeeper must play at least one outfield player period.
		-Direct headers are prohibited.
		-The distance between defenders and the offside line is increased for goal kicks.

**Table 2:** Cinematic variables studied.

Variables	Definition	Units
Total distance	Distance covered during the effective playing time	Metres
Distance in speed sections	Distance covered in relative speed segments during effective playing time	Metres
Number of accelerations	Number of accelerations during the effective playing time	Count
Accelerations in speed sections	Number of accelerations in relative speed segment during effective playing time	Count
Number of decelerations	Number of decelerations in relative speed segments during the effective playing time	Count
Decelerations in speed sections	Number of decelerations in relative speed segment during effective playing time	Count
Distance in acceleration	Distance covered in accelerations in relative speed sections during the effective playing time	Metres
Deceleration distance	Distance covered in decelerations in relative speed sections during the effective playing time	Metres
Maximum acceleration speed	Maximum acceleration speed achieved during the effective playing time	m/s^2^
Maximum deceleration speed	Maximum deceleration velocity achieved during the effective playing time	m/s^2^
Maximum speed	Maximum speed recorded during the effective playing time	km/h
Player Load	Player load during effective playing time in tis 3 axes (cranial–caudal, dorsal–ventral, and lateral–medial). It was calculated with the following equation: $\sqrt{\frac{{\left(A_{y^{1}}-A_{y^{-1}}\right)}^{2}+{\left(A_{X^{1}}-A_{X^{-1}}\right)}^{2}+{\left(A_{Z^{1}}-A_{Z^{-1}}\right)}^{2}}{100}\;\;}$	Arbitrary units

**Table 3:** Defined cut-off points for distance in speed, acceleration, and deceleration sections.

		Zone 1	Zone 2	Zone 3	Zone 4
Distance in speed sections (km/h)	Start	1	4.55	8.35	12.38
	End	4.55	8.35	12.38	∞
Acceleration (m/s^2^)	Start	0	2.93	7	
	End	2.93	6.99	∞	
Deceleration (m/s^2^)	Start	−0.83	−2.12	−2.12	
	End	0	−0.83	∞	

**Table 4:** Normality test results (Shapiro–Wilk).

Variable	*p*-Value	Normality
Total distance	0.779	Normal
Distance covered [1–4.552 km/h]	0.101	Normal
Distance covered [4.553–8.35 km/h]	0.697	Normal
Distance covered [8.351–12.377 km/h]	0.981	Normal
Distance covered [>12.378 km/h]	0.223	Normal
Number of accelerations	0.748	Normal
Number of decelerations	0.376	Normal
Distance covered in accelerating in zone 1	0.433	Normal
Distance covered in accelerating in zone 2	0.003	No normal
Distance covered in accelerating in zone 3	<0.001	No normal
Distance covered in decelerating in zone 1	0.663	Normal
Distance covered in decelerating in zone 2	0.412	Normal
Distance covered in decelerating in zone 3	0.120	Normal
Player load	0.576	Normal
Maximum acceleration speed	0.500	Normal
Maximum deceleration speed	0.005	No normal
Maximum speed	0.973	Normal

**Table 5:** Differences in normal cinematic variables between tournaments.

	T1		T2		*p*-Value	BF^p^_10_	Effect Size
	N	Mean ± SD	N	Mean ± SD			
Total distance	45	7692 ± 3190	45	6450 ± 1715	0.015 *	2.78	0.378
Distance covered [1–4.552 km/h]	45	2698 ± 1111	45	2547 ± 781	0.463	0.209	0.110
Distance covered [4.553–8.35 km/h]	45	2258 ± 912	45	2067 ± 622	0.185	0.375	0.201
Distance covered [8.351–12.377 km/h]	45	1709 ± 880	45	1501 ± 537	0.086	0.666	0.262
Distance covered [>12.378 km/h]	45	973 ± 681	45	880 ± 451	0.229	0.323	0.182
Number of accelerations	45	4099 ± 1851	45	3652 ± 986	0.193	0.364	0.197
Number of decelerations	45	3910 ± 1534	45	3642 ± 983	0.366	0.239	0.136
Player load	45	135 ± 68	45	130 ± 32	0.564	0.190	0.086
Maximum acceleration speed	45	13 ± 3	45	7.82 ± 2	<0.001 *	>100	1.604
Maximum speed	45	24 ± 4	45	25 ± 5	0.153	0.429	0.271
Distance covered accelerating in zone 1	45	3413 ± 1425	45	3060 ± 908	0.112	0.541	0.241
Distance covered decelerating in zone 1	45	466 ± 465	45	1343 ± 428	<0.001 *	>100	1.409
Distance covered decelerating in zone 2	45	516 ± 498	45	1306 ± 374	<0.001 *	>100	1.401
Distance covered decelerating in zone 3	45	634 ± 416	45	441.9 ± 209	<0.001 *	49.735	0.555

SD: standard deviation. *: significant differences.

**Table 6:** Differences in distance travelled in non-parametric cinematic variables.

	T1		T2		*p*-Value	BF^p^_10_	Effect Size
	N	Median (IQR)	N	Median (IQR)			
Maximum deceleration speed	45	−5.34 (0.2)	45	−9.48 (190)	<0.001	>100	1
Acceleration distance in zone 2	45	342 (188)	45	199 (174)	0.174	0.211	0.235
Acceleration distance in zone 3	45	190 (342)	45	5.68 (24.6)	<0.001	>100	0.816

IQR: interquartile range.

## Data Availability

Data is contained within the article.
